# Lower limb sagittal kinematic and kinetic modeling of very slow walking for gait trajectory scaling

**DOI:** 10.1371/journal.pone.0203934

**Published:** 2018-09-17

**Authors:** Andrew J. J. Smith, Edward D. Lemaire, Julie Nantel

**Affiliations:** 1 Ottawa Hospital Research Institute, Ottawa, Canada; 2 University of Ottawa, Department of Human Kinetics, University of Ottawa, Ottawa, Canada; 3 Faculty of Medicine, University of Ottawa, Ottawa, Canada; Northwestern University, UNITED STATES

## Abstract

Lower extremity powered exoskeletons (LEPE) are an emerging technology that assists people with lower-limb paralysis. LEPE for people with complete spinal cord injury walk at very slow speeds, below 0.5m/s. For the able-bodied population, very slow walking uses different neuromuscular, locomotor, postural, and dynamic balance control. Speed dependent kinetic and kinematic regression equations in the literature could be used for very slow walking LEPE trajectory scaling; however, kinematic and kinetic information at walking speeds below 0.5 m/s is lacking. Scaling LEPE trajectories using current reference equations may be inaccurate because these equations were produced from faster than real-world LEPE walking speeds. An improved understanding of how able-bodied people biomechanically adapt to very slow walking will provide LEPE developers with more accurate models to predict and scale LEPE gait trajectories. Full body motion capture data were collected from 30 healthy adults while walking on an instrumented self-paced treadmill, within a CAREN-Extended virtual reality environment. Kinematic and kinetic data were collected for 0.2 m/s—0.8 m/s, and self-selected walking speed. Thirty-three common sagittal kinematic and kinetic gait parameters were identified from motion capture data and inverse dynamics. Gait parameter relationships to walking speed, cadence, and stride length were determined with linear and quadratic (second and third order) regression. For parameters with a non-linear relationship with speed, cadence, or stride-length, linear regressions were used to determine if a consistent inflection occurred for faster and slower walking speeds. Group mean equations were applied to each participant’s data to determine the best performing equations for calculating important peak sagittal kinematic and kinetic gait parameters. Quadratic models based on walking speed had the strongest correlations with sagittal kinematic and kinetic gait parameters, with kinetic parameters having the better results. The lack of a consistent inflection point indicated that the kinematic and kinetic gait strategies did not change at very slow gait speeds. This research showed stronger associations with speed and gait parameters then previous studies, and provided more accurate regression equations for gait parameters at very slow walking speeds that can be used for LEPE joint trajectory development.

## Introduction

Motor adaptation to different gait speeds are relevant to lower extremity powered exoskeletons (LEPE) since predefined gait control strategies are typically used for persons with complete paraplegia [1]. Consistent with patients receiving neurological rehabilitation [2], persons using a LEPE walk at speeds between 0.1m/s and 0.55 m/s [3–13], with an average speed of 0.26 m/s [14]. However, LEPE predefined joint trajectories are typically developed from able-bodied individuals walking within a normal range of walking speeds. Since walking slowly is considered to be more complex [15] and uses different locomotor and postural control strategies [2,16,17], LEPE may be improved with predefined joint trajectories based on speed-appropriate slow gait biomechanics.

Despite a wealth of biomechanics literature on a range of gait speeds [2,18–27], the slowest walking speed in studies that predicted kinematic and kinetic parameters was 0.5 m/s, and averaged greater than 0.9 m/s. From some of these works, kinematic peak sagittal parameters were found to be positively correlated with gait speed, but that correlation coefficients from simple linear (R^2^<0.60) and quadratic (R^2^<0.45) regressions were weak [20,27]. As well, kinematics were significantly less accurate when calculated from regression equations produced from gait speeds outside those being modeled [27]. Contrary to kinematics, gait kinetics have shown strong relationships with gait speed [23,28], with correlation coefficients greater than 0.90 for knee flexion [28] and extension [20] moments. However, if regression equations are inaccurate at walking speeds outside the range they were produced from, even highly correlated kinetic equations may be inaccurate at very slow walking speeds.

The reasons for kinematics having lower correlations than kinetics may be gait speed variability and experimental methods [29,30]. An inherent problem with interpreting biomechanical results is that gait variable differences can often be partially or entirely explained by speed [30]. One method for controlling speed mediated effects on gait is the use of an instrumented treadmill to reduce outcome measure variability when researching task specific biomechanics [28,31]. However, treadmills that dictate constant walking speeds by reducing variability compared to over ground walking [32] may not reflect the joint’s natural mechanical environment [30]. However, this methodology would be sufficient for modeling kinematic and kinetic speed dependent changes in gait for LEPE development because a LEPE also imposes a consistent and less variable walking pattern. Recently, we assessed extremely slow walking speeds of abled bodied adults to determine if changes in strategy were required at LEPE walking speeds [33]. A consistent inflection point at 0.5 m/s was found for step time, stance time, and double support time, suggesting a change in strategy at very slow speeds that favours increased ground contact time. The effect of these slow walking speeds on common sagittal plane kinematic and kinetic parameters has yet to be determined.

The primary goal of this research was to produce a set of reference equations derived from very slow gait speeds to improve modelling accuracy of peak sagittal gait parameters for gait trajectory scaling and LEPE development. This research included very slow walking speeds that are common for exoskeleton users. Since gait speed is the product of cadence and stride length, we examined these three stride parameters for their relationship with sagittal kinematic and kinetic gait parameters. The research outcomes determined which stride parameter had the best relationships between very slow walking and peak sagittal kinetics and kinematics. Based on previous literature, we hypothesized that kinetics would have stronger associations with temporal spatial parameters. From our previous research on stride parameters [33], we hypothesised that a change in gait strategy would occur at 0.5m/s, indicated by an inflection point for parameters with non-linear relationships with speed, cadence, or stride-length. An improved understanding of gait kinematics and kinetics at speeds achievable by exoskeleton device users, by identifying how able-bodied people biomechanically adapt to very slow gait speeds, will provide LEPE developers with better models for predicting and scaling exoskeleton gait trajectories.

## Materials and methods

### Participants

Thirty able-bodied (AB) volunteers were recruited from staff, students, and volunteers at The Ottawa Hospital Rehabilitation Centre and University of Ottawa (15 males, 15 females; mass = 75.8±13.2 kg, height = 1.73±0.12 m; age = 30±10 years). To be enrolled in the study participants did not have health issues that would affect walking on a treadmill. Prior to testing, volunteers were notified of potential risks of participating in this research and signed an informed consent form. This study, including consent forms, was approved by both the Ottawa Health Science Network and the University of Ottawa Research Ethics Boards.

### Equipment

The CAREN-Extended virtual environment (Motekforce Link, Amsterdam, NL) was used for the movement activities and data collection. This system included 3D motion capture (Vicon, Oxford, UK), six degree of freedom (6-DOF) moving platform with an embedded dual-track treadmill (Bertec Corp., Columbus, OH) with force plates under each track sampled at 1000 Hz, 180° screen for 3D virtual world projection. Full body kinematics were tracked at 100 Hz, using a 6-DOF, 57-markerset [31].

### Procedure

Participant’s height, weight, and leg dominance were collected. Height and weight were used to scale the biomechanical model to each participant for three dimensional motion analysis. Leg dominance was determined by the participants answer to “what leg would you use to kick a ball as far as possible”. Participants were given time to acclimate to the seven slow walking speeds (0.2–0.8 m/s, incremented by 0.1) and to self-pace treadmill walking [32]. Participants walked 40 meters at each walking speed (total 320 m), through a virtual park scene that provided realistic optic flow. At least 10 successful left and right strides of level walking where the participant cleanly contacted the two force plates with their right and left feet were collected for each speed. Walking speeds were randomised to avoid learning bias.

### Data analysis

Three-dimensional marker data were filtered with a 4^th^ order, low pass Butterworth filter (10Hz). A 10-segment model was defined using Visual3D (C-Motion) [33,34] scaled to the participant's height and weight. Ground reaction force data were filtered with a zero lag Butterworth filter with a cut off frequency of 20 Hz. Matlab software (2016a, Mathworks, Matwick, MA) was used to identify 33 common peak sagittal kinematic and kinetic parameters (Table 1). Repeated measures analysis of variance (ANOVA) was performed to determine if leg dominance had an effect on very slow sagittal gait kinematics and kinetics, with a p <0.05 considered to be statistically significant.

**Table 1:** Peak sagittal kinematic and kinetic gait parameters.

Header	Parameter	Description
AAx1	Ankle Angle	Plantarflexion during early stance
AAx2	Ankle Angle	Dorsiflexion during stance
AAx3	Ankle Angle	Plantarflexion during swing
AAx4	Ankle Angle	Dorsiflexion during swing
AAxRG	Ankle Angle	Ankle range
KAx1	Knee Angle	Knee flexion at initial contact
KAx2	Knee Angle	Knee flexion during early stance
KAx3	Knee Angle	Knee extension during stance
KAx4	Knee Angle	Knee flexion during swing
KAxRG	Knee Angle	Knee range
HAx1	Hip Angle	Hip flexion during early stance
HAx2	Hip Angle	Hip extension during mid to late stance
HAx3	Hip Angle	Hip flexion during swing
HAxRG	Hip Angle	Hip range
AMx1	Ankle Mom	Dorsiflexor moment during early stance
AMx2	Ankle Mom	Plantarflexor moment during stance
KMx1	Knee Mom	Knee flexor moment just after initial contact
KMx2	Knee Mom	Knee extensor moment during early stance
KMx3	Knee Mom	Knee flexor moment during mid- late stance
KMx4	Knee Mom	Knee extensor moment during late stance
HMx1	Hip Mom	Hip extensor moment during stance
HMx2	Hip Mom	Hip flexor moment during stance
HMx3	Hip Mom	Hip extensor moment during swing
APx1	Ankle Power	Ankle power absorption during initial loading
APx2	Ankle Power	Ankle power absorption during mid-late stance
APx3	Ankle Power	Ankle power gen during stance
KPx1	Knee Power	1st generation power during early stance
KPx2	Knee Power	1st absorption power during early stance
KPx3	Knee Power	2nd generation power after loading response
KPx4	Knee Power	2nd absorption power during late stance
HPx1	Hip Power	Hip generation power during early stance
HPx2	Hip Power	Hip absorption power during late stance
HPx3	Hip Power	Hip generation power during late stance

Group means and standard deviations for each parameter were calculated at each speed. Linear and quadratic (second and third order) regressions were calculated to determine group mean equations for each of 33 sagittal gait parameters and 3 stride parameters (speed, cadence, stride-length). Pearson correlations were applied to determine the strength of association between each stride parameter and mean peak sagittal gait parameters. Correlation coefficients (R^2^) greater than 0.90 were considered strong, 70–89 moderate, 40–69 weak, and <39 poor.

For parameters with R^2^<0.9, linear regressions between each sagittal gait parameter and speed, cadence, or stride-length were used to determine if a consistent inflection point occurred for faster and slower walking speeds. For each parameter, linear regressions were performed for the following six gait speed sets (m/s): SP, 0.8, 0.7; SP, 0.8, 0.7, 0.6; SP, 0.8, 0.7, 0.6, 0.5; SP, 0.8, 0.7, 0.6, 0.5, 0.4; SP, 0.8, 0.7, 0.6, 0.5, 0.4, 0.3; SP, 0.8, 0.7, 0.6, 0.5, 0.4, 0.3, 0.2. If the correlation coefficients from a parameter’s speed sets dropped and remained below 0.90 for subsequent sets, a non-linear change was identified. An inflection point was identified if a non-linear change occurred consistently at the same speed for greater than 50% of participants.

The group mean equations were applied to each participant’s data to assess how well the equations represented the participant’s peak sagittal kinematics and kinetics. The best performing equations (i.e., individual correlation coefficients) fit the largest number of participants with a correlation coefficient greater than 0.90 and had the highest average correlation coefficient. If correlation coefficients were equally as high for the same number of study participants, the simpler equation was chosen.

To determine the difference between regression equations calculated outside the speed range of exoskeleton gait and our regression equations that were within the range, the predicted range between sagittal kinematics and kinetic parameters at 0.2m/s and 0.8m/s were computed using the best performing equations from Table 2 and 24 corresponding regression equations published by Lelas et al. [20]. The difference in the predicted range and difference as a percentage of the maximal range were compared between the two studies.

**Table 2:** Maximum sagittal plane kinematics and kinetics parameter regression equations. Best performing equations are bolded.

Parameter	Peak	Linear Equation	R^2^	Quadratic Equation 2nd Order	R^2^	Quadratic Equation 3rd Order	R^2^
**Ankle Angle**	**AAx1**	**y = -0.69*s*–7.45**	0.38	y = 5.45*s*^2^–9.13*s*–4.97	0.30	y = -8.47*s*^3^ + 24.47*s*^2^–20.93*s*–2.99	0.30
	AAx2	y = 1.24*s* + 11.79	0.32	**y = -6.47*s***^**2**^ **+ 11.25*s* + 8.84**	0.57	y = 1.46*s*^3^–9.74*s*^2^ + 13.28*s* + 8.5	0.57
	AAx3	y = -12.16*s*–3.14	0.76	y = 0.08*s*^2^–12.28*s*–3.1	0.76	**y = 3.2*s***^**3**^**–7.11*s***^**2**^**–7.82*s*–3.85**	0.76
	AAx4	y = -2.49*s* + 4.67	0.46	**y = 7.44*s***^**2**^**–14.01*s* + 8.07**	0.56	y = 6.03*s*^3^–6.1*s*^2^–5.61*s* + 6.66	0.57
	AAxRG	y = 9.63*s* + 19.57	0.72	**y = -4.25*s***^**2**^ **+ 16.21*s* + 17.63**	0.73	y = 3.66*s*^3^–12.47*s*^2^ + 21.31*s* + 16.77	0.73
**Knee Angle**	KAx1	y = -1.07*s* + 2.08	0.35	**y = 10.19*s***^**2**^**–16.84*s* + 6.73**	0.39	y = -12.77*s*^3^ + 38.87*s*^2^–34.64*s* + 9.71	0.41
	KAx2	y = 10.21*s* + 1.63	0.68	**y = 9.91*s***^**2**^**–5.13*s* + 6.15**	0.72	y = -22.79*s*^3^ + 61.07*s*^2^–36.88*s* + 11.46	0.72
	KAx3	y = 2.89*s*–1.02	0.51	y = 0.38*s*^2^ + 2.3*s*–0.85	0.51	**y = -3.4*s***^**3**^ **+ 8.01*s***^**2**^**–2.43*s*–0.05**	0.52
	KAx4	y = 13.37*s* + 47.39	0.77	**y = -11.77*s***^**2**^ **+ 31.59*s* + 42.02**	0.84	y = -4.54*s*^3^–1.57*s*^2^ + 25.26*s* + 43.08	0.84
	KAxRG	y = 14.88*s* + 49.25	0.76	**y = -16.35*s***^**2**^ + 40.19*s* + 41.8*	0.88	y = -5.85*s*^3^–3.22*s*^2^ + 32.04*s* + 43.16	0.88
**Hip Angle**	HAx1	y = 6.97*s* + 12.52	0.81	**y = -0.62*s***^**2**^ + 7.92*s* + 12.24*	0.81	y = 2.13*s*^3^–5.4*s*^2^ + 10.89*s* + 11.75	0.81
	**HAx2**	y = -7.46*s*–7.17*	0.85	y = -2.14*s*^2^–4.14*s*–8.14	0.85	y = -1.58*s*^3^ + 1.4*s*^2^–6.34*s*–7.78	0.85
	**HAx3**	**y = 6.03*s* + 15.94**	0.75	y = -3.01*s*^2^ + 10.69*s* + 14.57	0.74	y = -5.97*s*^3^ + 10.39*s*^2^ + 2.37*s* + 15.96	0.74
	HAxRG	y = 13.51*s* + 23.22*	0.92	y = -0.79*s*^2^ + 14.74*s* + 22.85	0.92	y = -4.09*s*^3^ + 8.39*s*^2^ + 9.04*s* + 23.81	0.92
**Ankle Moment**	AMx1	y = -0.16*s*–0.01	0.89	**y = -0.01*s***^**2**^–0.14*s*–0.02*	0.89	y = -0.01*s*^3^ + 0.01*s*^2^–0.15*s*–0.01	0.89
	AMx2	y = 0.71*s* + 0.60	0.93	**y = -0.22*s***^**2**^ + 1.04*s* + 0.5*	0.95	y = -0.03*s*^3^–0.14*s*^2^ + 1*s* + 0.51	0.95
**Knee Moment**	KMx1	**y = -0.17*s*–0.06**	0.78	y = 0.02*s*^2^–0.21*s*–0.05	0.78	y = 0.09*s*^3^–0.17*s*^2^–0.09*s*–0.07	0.78
	KMx2	y = 0.54*s*–0.13	0.84	**y = 0.35*s***^**2**^ + 0*s* + 0.03*	0.89	y = -0.32*s*^3^ + 1.07*s*^2^–0.45*s* + 0.11	0.89
	KMx3	y = -0.12*s*–0.20	0.53	**y = -0.07*s***^**2**^ **+ 0*s*–0.23**	0.55	y = -0.12*s*^3^ + 0.19*s*^2^–0.17*s*–0.2	0.55
	KMx4	y = 0.10*s* + 0.04	0.72	**y = 0.03*s***^**2**^ **+ 0.06*s* + 0.05**	0.73	y = -0.1*s*^3^ + 0.25*s*^2^–0.08*s* + 0.08	0.73
**Hip Moment**	HMx1	y = 0.59*s* + 0.01	0.92	**y = 0.24*s***^**2**^ + 0.22*s* + 0.11*	0.94	y = -0.15*s*^3^ + 0.57*s*^2^ + 0.02*s* + 0.15	0.94
	HMx2	y = -0.53*s*–0.07*	0.95	y = -0.14*s*^2^–0.31*s*–0.14	0.96	y = -0.02*s*^3^–0.09*s*^2^–0.34*s*–0.13	0.96
	HMx3	y = 0.31*s*–0.03*	0.93	y = 0.07*s*^2^ + 0.19*s* + 0.01	0.93	y = -0.17*s*^3^ + 0.46*s*^2^–0.05*s* + 0.05	0.93
**Ankle Power**	APx1	y = -0.45*s* + 0.11	0.90	**y = -0.31*s***^**2**^ + 0.02*s*–0.03*	0.96	y = -0.21*s*^3^ + 0.16*s*^2^–0.27*s* + 0.01	0.96
	APx2	y = -0.62*s*–0.19	0.71	y = 0.79*s*^2^–1.84*s* + 0.17*	0.88	**y = 0.82*s***^**3**^**–1.06*s***^**2**^**–0.69*s*–0.02**	0.89
	APx3	y = 3.23*s*–0.69*	0.96	y = 1.12*s*^2^ + 1.49*s*–0.17	0.98	y = -1.91*s*^3^ + 5.41*s*^2^–1.16*s* + 0.27	0.98
**Knee Power**	KPx1	y = 0.47*s*–0.14	0.85	**y = 0.29*s***^**2**^ + 0.02*s*–0.01*	0.89	y = -0.38*s*^3^ + 1.14*s*^2^–0.51*s* + 0.08	0.90
	KPx2	y = -0.8*s* + 0.26	0.81	**y = -0.8*s***^**2**^ + 0.44*s*–0.11*	0.93	y = -0.07*s*^3^–0.65*s*^2^ + 0.34*s*–0.09	0.93
	KPx3	y = 0.48*s*–0.02	0.87	y = 0.12*s*^2^ + 0.3*s* + 0.03	0.87	**y = 0.02*s***^**3**^ **+ 0.08*s***^**2**^ + 0.33*s* + 0.03*	0.88
	KPx4	y = -0.78*s* + 0.09	0.92	**y = -0.27*s***^**2**^–0.37*s*–0.04*	0.93	y = 0.2*s*^3^–0.71*s*^2^–0.09*s*–0.08	0.93
**Hip Power**	HPx1	y = 0.58*s*–0.06	0.84	**y = 0.14*s***^**2**^ + 0.36*s* + 0*	0.84	y = 0.05*s*^3^ + 0.04*s*^2^ + 0.43*s*–0.01	0.84
	HPx2	y = -0.47*s* + 0.09	0.90	**y = -0.34*s***^**2**^ + 0.05*s*–0.06*	0.96	y = -0.27*s*^3^ + 0.26*s*^2^–0.32*s* + 0	0.96
	HPx3	y = 0.74*s*–0.08	0.95	y = 0.19*s*^2^ + 0.44*s* + 0.01	0.95	**y = -0.25*s***^**3**^ **+ 0.76*s***^**2**^ + 0.09*s* + 0.07*	0.96

* Variables where more than 50% of samples had a correlation coefficient > 0.9. Gait speed (*s*)

## Results

At each speed interval, an average of 24 ± 8 steps were analyzed. Thirty-three common sagittal parameters were evaluated at the ankle, knee, and hip from the last 10 successful steps (Table 1). Peaks included 14 kinematic measures (joint angles, ranges) and 19 kinetic measures (joint moments, powers). No significant differences were observed between dominant and non-dominant limbs, therefore only the dominant limb was used for analysis.

From Pearson correlations of group mean data, gait speed had stronger correlations than stride-length and cadence for 18 of 33 parameters (KAx2, KAx4, HAx2, HAx3, KMx1, KMx2, KMx3, HMx1, HMx2, HMx3, APx1, APx3, KPx1, KPx3, KPx4, HPx1, HPx2, HPx3). Stride-length had the strongest association with five parameters (AAx3, AAxRG, AMx1, AMx2, KMx4), and cadence only two parameters (KAxRG, APx2). Hip flexion during early stance (HAx1) and hip range of motion (HAxRG) were associated equally with gait speed and stride-length. For all 33 parameters, correlation coefficients were highest using second order quadratic equations. No consistent point of inflection was identified for any sagittal gait parameter.

From Pearson correlations of group mean regression equations fit to individual participant data, the same 12 sagittal gait parameters (AMx2, HMx1, APx1, KPx1, KPx2, KPx4, HPx2, HAxRG, HMx2, HMx3, APx3, HPx3) had strong associations with cadence, gait speed, and stride-length. Gait speed had the strongest associations, thus only results for speed were reported in Table 2. Equations for cadence and stride-length can be found in supporting information (S1 and S2 Tables). Of the 12 strongly correlated parameters for gait speed, all but one (HAxRG) were a kinetic parameter and most were best fit using a second order quadratic (AMx2, HMx1, APx1, KPx1, KPx2, KPx4, HPx2). Linear equations strongly predicted HAxRG, HMx2, HMx3, and APx3 while third order quadratic formulas strongly fit the kinetic parameter HPx3.

Parameters with moderate correlation coefficients (0.7<R^2^<0.9) that fit at least 50% of participants with individual correlation coefficients > 0.90 were: KAxRG, HAx1, and HAx2 (kinematic parameters) and AMx1, KMx2, APx2, KPx3, and HPx1 (kinetic parameters). Moderate correlation coefficients that fit less than 50% of participants were AAx3, AAxRG, KAx2, KAx4, HAx3 (kinematic parameters) and KMx1 and KMx4 (kinetic parameters). Weak and poor correlation coefficients were found for kinematic parameters AAx1, AAx2, AAx4, KAx1, KAx3 and kinetic parameter KMx3.

Of 24 corresponding sagittal gait parameter regression equations reported by Lelas et al. (Table 3), 14 corresponded with the best performing equation types reported in Table 2. Excluding hip extension moment and hip power generation during loading response, gait parameters from Lelas et al. equations were all overestimated. Predicted range of peak knee joint angles during stance and peak ankle plantarflexion angle differed by more than 5°, and by as much as 10.57°. Range of peak knee flexion moment during loading response and pre-swing, as well as peak ankle dorsiflexion moment predicted by Lelas et al., were more the 62% greater than values predicted using our equations. Joint power was overestimated by at least 58.8% for hip power generation during pre-swing, knee absorption during loading response, and ankle peak absorption.

**Table 3:** Predicted range (0.2m/s to 0.8m/s) of sagittal kinematic and kinetic variables using the best equations from Table 2 and Lelas et al. (2003). Differences and differences as a percent of the maximum variable were between our study and Lelas et al.

Peak Sagittal Gait Parameter	Units	Reg: Table 2		Lelas et al.		Difference	% of Max Range
		Range	Reg	Range	Reg		
Hip flexion	Degrees	4.38	(Q2)	4.43	(L)	0.05	1.1
Hip extension	Degrees	4.48	(L)	3.07	(L)	1.41	31.5
Knee extension at initial contact	Degrees	3.99	(Q2)	12.05	(Q2)	8.06	66.9
Knee flexion loading response	Degrees	2.89	(Q2)	13.46	(Q2)	10.57	78.6
Knee extension terminal stance	Degrees	1.63	(Q3)	9.96	(Q2)	8.33	83.6
Knee flexion swing	Degrees	11.89	(Q2)	7.04	(Q2)	4.85	40.8
Ankle plantar flexion loading response	Degrees	0.41	(L)	1.06	(L)	0.64	60.8
Ankle dorsiflexion mid stance	Degrees	2.87	(Q2)	1.44	(L)	1.43	49.8
Ankle plantarflexion	Degrees	7.35	(Q3)	2.27	(L)	5.08	69.1
Ankle dorsiflexion swing	Degrees	3.94	(Q2)	3.95	(Q2)	0.01	0.3
Hip flexion moment	Nm	23.95	(L)	24.94	(Q2)	0.99	4.0
Hip extension moment	Nm	20.78	(Q2)	20.26	(Q2)	-0.52	-2.5
Knee flexion moment loading response	Nm	7.68	(L)	25.72	(L)	18.04	70.1
Knee extension moment terminal Stance	Nm	4.07	(Q2)	3.90	(L)	-0.17	-4.2
Knee flexion moment pre-swing	Nm	3.16	(Q2)	11.69	(L)	8.53	72.9
Ankle dorsiflexion moment	Nm	6.78	(Q2)	17.93	(L)	11.15	62.2
Hip power generation loading response	W	22.59	(Q2)	10.91	(Q2)	-11.68	-51.7
Hip power absorption	W	13.10	(Q2)	20.95	(Q2)	7.85	37.5
Hip power generation pre-swing	W	28.92	(Q3)	70.14	(Q2)	41.23	58.8
Knee power absorption loading response	W	16.26	(Q2)	97.42	(Q2)	81.15	83.3
Knee power generation mid-stance	W	19.28	(Q3)	19.87	(L)	0.59	3.0
Knee power absorption pre-swing	W	28.92	(Q2)	32.73	(Q2)	3.82	11.7
Ankle power absorption	W	47.95	(Q3)	125.48	(Q3)	77.53	61.8
Ankle power generation pre-swing	W	145.93	(L)	176.13	(L)	30.20	17.1

(Reg) Regression type: (L) linear, (Q2) second order Quadratic, (Q3) third order quadratic.

## Discussion

The primary goal of this study was to provide LEPE developers with equations for modelling speed related changes in sagittal peak joint kinematics and kinetics. These peaks could then be used to more appropriately scale predefined LEPE joint trajectories. Appropriately scaled trajectories may enhance LEPE function, making it easier for users to complete steps successfully [11], enhancing mobility, balance, cadence, and walking speed of people with complete lower limb paralysis. This study compiled a comprehensive reference data set of 33 peak sagittal kinematic and kinetic parameters at very slow gait speeds that have previously received little attention in the literature.

The strongest regression equations were between peak kinetics and gait speed. When fit to participant data, these equations produced correlation coefficients much higher than previously reported. For example, hip extension moment was reported to have a second order quadratic relationship with gait speed, with correlation coefficients ranging between 0.72 and 0.89 [20,28,34]. We found that peak hip extension moments in early stance and late swing were best fit using second order quadratic and linear regressions, with both higher regression correlation coefficients (0.94 and 0.93) and individually fitted coefficients (87% and 90%). The strength of correlations in this study may be due to our use of an instrumented treadmill which may reduce outcome measure variability [27,30,35].

The main limitation to this study was how to control walking speeds. A treadmill was used rather than vague instructions (e.g., “walk fast”, “walk slow”) that can result in an unbalanced dataset where participant may not walk at a given speed for an equal number of stride [24]. Treadmill studies offer the ability to collect numerous consecutive strides with greater reproducibility and reduce stride-length variability [25]. The number of consecutive strides and reduced gait variability associated with fixed-speed treadmill use may explain why our regression values were greater than those previously reported; however, treadmill use may have influenced gait parameters [36] by shortening stride length and cadence, increasing knee extension and forward trunk lean through stance, and increasing hip and knee flexion through swing [37,38]. If data from this studies is used for clinical decision making on overground walking, the potential for less variability in the treadmill data should be considered. However, since LEPE impose consistent and less variable walking patterns, treadmill gait is appropriate for developing joint trajectories for powered exoskeleton devices. LEPE stride parameter variability can occur due to early foot strikes and varying step initiation timing, which are independent of preset joint trajectories. Therefore, research on short step correction control is also needed for safe and efficient device use.

Like our results and previous studies [20,23,28], sagittal kinematic and kinetic parameters correlated with speed, but kinematic parameters had poorer correlations. However, regression types were not always consistent with our results. Of 24 regression equations for peak sagittal kinematics, reported by Lelas et al. (Table 3), only 14 were consistent with regression equations types in our research. As well, the range of calculated peak kinematics and kinetics between 0.2 m/s and 0.8 m/s differed between our results and Lelas et al. [20]. Lelas et al. produced regression equation at 0.5m/s, which was nearly twice the average LEPE user walking speed. Kinematic and kinetic regression equations from similar studies can be inaccurate at speeds achievable by a LEPE user [27].

Though kinematic parameters had lower correlations, speed associations in this study were much stronger than correlation results in the literature. Lelas et al. [20] reported a poor linear relationship (R^2^ = 0.14) for gait speed and peak hip extension (HAx2) during stance. Our results produced an average correlation coefficient of 0.85. As well, knee flexion during loading response (KAx2) and swing (KAx4) had weak relationships with speed (R^2^ = 0.60 and 0.43, respectively) in the Lelas et al. study. Our results supported this quadratic relationship with speed but with moderate correlation coefficients of 0.72 for KAx2 and 0.84 for KAx4. Kinematic parameters had low correlation coefficients, likely due to the many degrees of freedom available to the lower limb when adapting to various very slow gait speeds [39,40]. Therefore gait trajectory choices will differ across people and walking scenarios.

Lower kinematic correlations (i.e., below 0.9) are supported by studies investigating how able bodied persons adapt to LEPE assisted gait [41–45]. With LEPE assistance, total ankle and hip moment (muscle plus exoskeleton) were almost identical to passive walking, with both walking scenarios producing large differences in joint angles and EMG patterns between LEPE assisted and control steps. Joint kinematic patterns may be less important to nervous system planning, with the lower limb adapting by prioritising kinetic optimisation [46], unlike the upper limb prioritises kinematic control during reaching [47–50]. Altering musculoskeletal mechanics by applying assistive forces results in variable kinematics and invariant moments of the lower limb, advancing our understanding of how the lower limb optimises motor adaptation. Kinetic parameters could also be used to predict exoskeleton mechanical output during different tasks, aiding robotic exoskeleton design.

## Conclusion

The goal of this research was to provide better equations for LEPE developers to determine appropriate peak sagittal kinematics and kinetics for joint trajectory development. Quadratic models based on walking speed had the strongest correlations with most peak sagittal kinematic and kinetic gait parameters, with kinetic parameters having the better results. This research showed that peak sagittal kinematic and kinetic gait parameters, between 0.2 and 0.8 m/s, had a strong non-linear association with speed. The lack of a consistent inflection point indicated that the gait kinematic and kinetic strategy did not change at very slow gait speeds. Inconsistent inflection points may demonstrate how individuals adapt to slow speeds differently. While these equations should be tested on a separate dataset, within the same gait speed range, equations produced in this research showed stronger associations with speed then previous studies. The regression equations defined in this research should provide better results when modeling LEPE joint trajectories at very slow walking speeds.

## Supporting information

S1 Table Maximum sagittal plane kinematics and kinetics parameter regression equations for cadence. Cadence (*c*).
(DOCX)

S2 Table Maximum sagittal plane kinematics and kinetics parameter regression equations for stride length. Stride length (*l*).
(DOCX)

S1 Supporting Data This excel document includes means and standard deviations for 33 peak sagittal gait parameters from the 30 able-bodied participants in this study. Each tab is labled with a gait parameter header defined within the Data Key tab. Data has been provided for both the dominant (Dom) and non-dominant limbs (NonD). Columns are mean peak data from level ground (LG) walking at 7 set waking speeds collected for 0.2 m/s—0.8 m/s, and a self-selected walking speed condition. Headers ending in "sd" contain the standard deviations for the for 33 peak sagittal gait parameters.
(XLSX)
