# Supplementary material for: Lower limb sagittal kinematic and kinetic modeling of very slow walking for gait trajectory scaling
Source: PLoS One. 2018 Sep 17;13(9):e0203934. doi: 10.1371/journal.pone.0203934 (PMC6141077; doi:10.1371/journal.pone.0203934)
Supplement: S1 Table — Cadence (c). (DOCX) [file pone.0203934.s001.docx]

**S1 Table:** Maximum sagittal plane kinematics and kinetics parameter regression equations for cadence.

| **Parameter** | **Peak** | **Linear Equation** | **R²** | **Quadratic Equation 2nd Order** | **R²** | **Quadratic Equation 3rd Order** | **R²** |
| --- | --- | --- | --- | --- | --- | --- | --- |
| **Ankle Angle** | AAx1 | y = 2.10*c* + -9.7 | 0.36 | y = 5.24*c*^2^ - 8.28*c* - 5.03 | 0.29 | y = -15.85*c*^3^ + 51.69*c*^2^ - 51.03*c* + 7.34 | 0.30 |
|  | AAx2 | y = -2.76*c* + 14.95 | 0.43 | y = -3.53*c*^2^ + 4.22*c* + 11.80 | 0.48 | y = 22.2*c*^3^ - 68.59*c*^2^ + 64.1*c* - 5.52 | 0.56 |
|  | AAx3 | y = 12.62*c* + -21.46 | 0.65 | y = -24.70*c*^2^ + 61.55*c* - 43.50 | 0.75 | y = 59.35*c*^3^ - 198.64*c*^2^ + 221.62*c* - 89.82 | 0.76 |
|  | AAx4 | y = 4.14*c* + -0.45 | 0.55 | y = 0.3*c*^2^ + 3.56*c* - 0.18 | 0.53 | y = -24.61*c*^3^ + 72.41*c*^2^ - 62.81*c* + 19.02 | 0.56 |
|  | AAxRG | y = -11.04*c* + 35 | 0.69 | y = 14.75*c*^2^ - 40.26*c* + 48.16 | 0.73 | y = -29.76*c*^3^ + 101.97*c*^2^ - 120.53*c* + 71.38 | 0.73 |
| **Knee**  **Angle** | KAx1 | y = 3.65*c* + -1.75 | 0.37 | y = 10.11*c*^2^ - 16.37*c* + 7.27 | 0.38 | y = -30.25*c*^3^ + 98.76*c*^2^ - 97.96*c* + 30.88 | 0.41 |
|  | KAx2 | y = -7.96*c* + 14.71 | 0.48 | y = 34.75*c*^2^ - 76.8*c* + 45.71 | 0.68 | y = -75.01*c*^3^ + 254.58*c*^2^ - 279.1*c* + 104.25 | 0.72 |
|  | KAx3 | y = -2.86*c* + 3.21 | 0.42 | y = 6.81*c*^2^ - 16.35*c* + 9.29 | 0.50 | y = -13.75*c*^3^ + 47.11*c*^2^ - 53.43*c* + 20.02 | 0.51 |
|  | KAx4 | y = -16.47*c* + 69.8 | 0.82 | y = 17.04*c*^2^ - 50.22*c* + 85 | 0.83 | y = -14.15*c*^3^ + 58.52*c*^2^ - 88.39*c* + 96.05 | 0.84 |
|  | KAxRG | y = -19.03*c* + 74.81 | 0.86 | y = 16.42*c*^2^ - 51.55*c* + 89.46 | 0.86 | y = 0.95*c*^3^ + 13.62*c*^2^ - 48.98*c* + 88.72 | 0.87 |
| **Hip**  **Angle** | HAx1 | y = -7.42*c* + 23.18 | 0.67 | y = 13.24*c*^2^ - 33.65*c* + 35 | 0.77 | y = -30.3*c*^3^ + 102.03*c*^2^ - 115.36*c* + 58.64 | 0.80 |
|  | HAx2 | y = 7.29*c* + -18.02 | 0.69 | y = -16.95*c*^2^ + 40.87*c* - 33.14 | 0.81 | y = 42.07*c*^3^ - 140.25*c*^2^ + 154.34*c* - 65.97 | 0.84 |
|  | HAx3 | y = -6.82*c* + 25.52 | 0.67 | y = 11.04*c*^2^ - 28.70*c* + 35.37 | 0.74 | y = -9.62*c*^3^ + 39.25*c*^2^ - 54.66*c* + 42.88 | 0.74 |
|  | HAxRG | y = -14.14*c* + 43.68 | 0.78 | y = 28.06*c*^2^ - 69.71*c* + 68.71 | 0.90 | y = -52.35*c*^3^ + 181.48*c*^2^ - 210.91*c* + 109.57 | 0.91 |
| **Ankle Moment** | AMx1 | y = 0.16*c* + -0.25 | 0.75 | y = -0.33*c*^2^ + 0.81*c* - 0.54 | 0.86 | y = 0.75*c*^3^ - 2.53*c*^2^ + 2.84*c* - 1.12 | 0.88 |
|  | AMx2 | y = -0.78*c* + 1.7 | 0.87 | y = 1.28*c*^2^ - 3.32*c* + 2.85 | 0.94 | y = -2.1*c*^3^ + 7.44*c*^2^ - 8.98*c* + 4.49 | 0.94 |
| **Knee**  **Moment** | KMx1 | y = 0.18*c* + -0.32 | 0.66 | y = -0.37*c*^2^ + 0.91*c* - 0.65 | 0.77 | y = 0.55*c*^3^ - 1.98*c*^2^ + 2.40*c* - 1.08 | 0.78 |
|  | KMx2 | y = -0.48*c* + 0.62 | 0.59 | y = 1.50*c*^2^ - 3.45*c* + 1.95 | 0.83 | y = -3.73*c*^3^ + 12.43*c*^2^ - 13.51*c* + 4.86 | 0.88 |
|  | KMx3 | y = 0.11*c* + -0.36 | 0.43 | y = -0.30*c*^2^ + 0.71*c* - 0.63 | 0.51 | y = 0.76*c*^3^ - 2.53*c*^2^ + 2.76*c* - 1.23 | 0.54 |
|  | KMx4 | y = -0.1*c* + 0.19 | 0.58 | y = 0.25*c*^2^ - 0.60*c* + 0.41 | 0.72 | y = -0.54*c*^3^ + 1.83*c*^2^ - 2.05*c* + 0.83 | 0.73 |
| **Hip**  **Moment** | HMx1 | y = -0.56*c* + 0.85 | 0.70 | y = 1.49*c*^2^ - 3.52*c* + 2.18 | 0.91 | y = -3.42*c*^3^ + 11.53*c*^2^ - 12.75*c* + 4.86 | 0.94 |
|  | HMx2 | y = 0.52*c* + -0.84 | 0.75 | y = -1.22*c*^2^ + 2.92*c* - 1.92 | 0.92 | y = 2.89*c*^3^ - 9.69*c*^2^ + 10.72*c* - 4.18 | 0.95 |
|  | HMx3 | y = -0.3*c* + 0.42 | 0.73 | y = 0.74*c*^2^ - 1.77*c* + 1.08 | 0.91 | y = -1.45*c*^3^ + 5.00*c*^2^ - 5.68*c* + 2.21 | 0.93 |
| **Ankle**  **Power** | APx1 | y = 0.40*c* + -0.52 | 0.62 | y = -1.19*c*^2^ + 2.77*c* - 1.58 | 0.88 | y = 3.27*c*^3^ - 10.78*c*^2^ + 11.59*c* - 4.14 | 0.95 |
|  | APx2 | y = 0.80*c* + -1.26 | 0.84 | y = -0.75*c*^2^ + 2.29*c* - 1.93 | 0.84 | y = -1.27*c*^3^ + 2.98*c*^2^ - 1.15*c* - 0.94 | 0.87 |
|  | APx3 | y = -3.07*c* + 3.94 | 0.73 | y = 8.10*c*^2^ - 19.12*c* + 11.16 | 0.95 | y = -17.89*c*^3^ + 60.52*c*^2^ - 67.36*c* + 25.12 | 0.98 |
| **Knee**  **Power** | KPx1 | y = -0.42*c* + 0.5 | 0.60 | y = 1.31*c*^2^ - 3.00*c* + 1.67 | 0.86 | y = -3.13*c*^3^ + 10.48*c*^2^ - 11.45*c* + 4.11 | 0.90 |
|  | KPx2 | y = 0.65*c* + -0.79 | 0.50 | y = -2.39*c*^2^ + 5.38*c* - 2.92 | 0.82 | y = 6.81*c*^3^ - 22.35*c*^2^ + 23.75*c* - 8.24 | 0.92 |
|  | KPx3 | y = -0.47*c* + 0.69 | 0.70 | y = 1.14*c*^2^ - 2.73*c* + 1.70 | 0.85 | y = -2.36*c*^3^ + 8.06*c*^2^ - 9.10*c* + 3.54 | 0.87 |
|  | KPx4 | y = 0.74*c* + -1.03 | 0.71 | y = -1.87*c*^2^ + 4.45*c* - 2.70 | 0.89 | y = 4.53*c*^3^ - 15.14*c*^2^ + 16.67*c* - 6.23 | 0.93 |
| **Hip**  **Power** | HPx1 | y = -0.57*c* + 0.79 | 0.68 | y = 1.37*c*^2^ - 3.28*c* + 2.01 | 0.82 | y = -2.92*c*^3^ + 9.93*c*^2^ - 11.16*c* + 4.29 | 0.84 |
|  | HPx2 | y = 0.42*c* + -0.56 | 0.62 | y = -1.23*c*^2^ + 2.86*c* - 1.66 | 0.87 | y = 3.6*c*^3^ - 11.80*c*^2^ + 12.58*c* - 4.48 | 0.96 |
|  | HPx3 | y = -0.72*c* + 1 | 0.75 | y = 1.76*c*^2^ - 4.20*c* + 2.56 | 0.92 | y = -3.99*c*^3^ + 13.45*c*^2^ - 14.97*c* + 5.68 | 0.95 |

Cadence (*c*)
