# Supplementary material for: Lower limb sagittal kinematic and kinetic modeling of very slow walking for gait trajectory scaling
Source: PLoS One. 2018 Sep 17;13(9):e0203934. doi: 10.1371/journal.pone.0203934 (PMC6141077; doi:10.1371/journal.pone.0203934)
Supplement: S2 Table — Stride length (l). (DOCX) [file pone.0203934.s002.docx]

**S2 Table:** Maximum sagittal plane kinematics and kinetics parameter regression equations for stride length.

| **Parameter** | **Peak** | **Linear Equation** | **R²** | **Quadratic Equation 2nd Order** | **R²** | **Quadratic Equation 3rd Order** | **R²** |
| --- | --- | --- | --- | --- | --- | --- | --- |
| **Ankle Angle** | AAx1 | y = -1.18*l* - 6.81 | 0.37 | y = 10.4*l*^2^ - 21.82*l* + 2.73 | 0.30 | y = -12.58*l*^3^+ 47.46*l*^2^ - 56.39*l* + 12.97 | 0.30 |
|  | AAx2 | y = 1.97*l* + 10.77 | 0.38 | y = -11.86*l*^2^ + 25.51*l* - 0.12 | 0.54 | y = -4.89*l*^3^+ 2.53*l*^2^ + 12.08*l* + 3.86 | 0.57 |
|  | AAx3 | y = -17.05*l* + 4.84 | 0.72 | y = -4.05*l*^2^ - 9.02*l* + 1.13 | 0.76 | y = 7.49*l*^3^- 26.09*l*^2^ + 11.55*l* - 4.96 | 0.75 |
|  | AAx4 | y = -3.74*l* + 6.53 | 0.51 | y = 13*l*^2^ - 29.54*l* + 18.46 | 0.52 | y = 23.49*l*^3^- 56.2*l*^2^ + 35.03*l* - 0.65 | 0.56 |
|  | AAxRG | y = 13.66*l* + 13.11 | 0.72 | y = -4.92*l*^2^ + 23.43*l* + 8.59 | 0.73 | y = 2.63*l*^3^- 12.67*l*^2^ + 30.65*l* + 6.45 | 0.73 |
| **Knee**  **Angle** | KAx1 | y = -1.88*l* + 3.13 | 0.36 | y = 19.44*l*^2^ - 40.46*l* + 20.97 | 0.41 | y = -17.58*l*^3^+ 71.22*l*^2^ - 88.77*l* + 35.27 | 0.41 |
|  | KAx2 | y = 13.93*l* - 4.72 | 0.58 | y = 23.13*l*^2^ - 31.97*l* + 16.51 | 0.72 | y = -42.48*l*^3^+ 148.25*l*^2^ - 148.71*l* + 51.07 | 0.72 |
|  | KAx3 | y = 4.04*l* - 2.9 | 0.47 | y = 1.81*l*^2^ + 0.44*l* - 1.24 | 0.52 | y = -7.61*l*^3^+ 24.22*l*^2^ - 20.47*l* + 4.95 | 0.52 |
|  | KAx4 | y = 19.15*l* + 38.25 | 0.82 | y = -17.63*l*^2^ + 54.12*l* + 22.08 | 0.83 | y = -24.98*l*^3^+ 55.95*l*^2^ - 14.53*l* + 42.4 | 0.84 |
|  | KAxRG | y = 21.42*l* + 38.99 | 0.84 | y = -25.72*l*^2^ + 72.45*l* + 15.39 | 0.85 | y = -34.27*l*^3^+ 75.21*l*^2^ - 21.73*l* + 43.26 | 0.87 |
| **Hip**  **Angle** | HAx1 | y = 9.78*l* + 7.94 | 0.76 | y = 1.14*l*^2^ + 7.52*l* + 8.98 | 0.80 | y = 3.22*l*^3^- 8.34*l*^2^ + 16.37*l* + 6.37 | 0.81 |
|  | HAx2 | y = -10.38*l* - 2.34 | 0.79 | y = -6.56*l*^2^ + 2.65*l* - 8.37 | 0.85 | y = -4.61*l*^3^+ 7.02*l*^2^ - 10.03*l* - 4.62 | 0.85 |
|  | HAx3 | y = 8.54*l* + 11.91 | 0.72 | y = -3.39*l*^2^ + 15.27*l* + 8.8 | 0.74 | y = -18.69*l*^3^+ 51.64*l*^2^ - 36.09*l* + 24 | 0.74 |
|  | HAxRG | y = 18.95*l* + 14.34 | 0.87 | y = 3.32*l*^2^ + 12.37*l* + 17.39 | 0.92 | y = -13.29*l*^3^+ 42.45*l*^2^ - 24.14*l* + 28.2 | 0.92 |
| **Ankle Moment** | AMx1 | y = -0.22*l* + 0.09 | 0.84 | y = -0.07*l*^2^ - 0.08*l* + 0.03 | 0.89 | y = -0.01*l*^3^- 0.05*l*^2^ - 0.09*l* + 0.03 | 0.89 |
|  | AMx2 | y = 1*l* + 0.13 | 0.93 | y = -0.17*l*^2^ + 1.33*l* - 0.03 | 0.94 | y = -0.51*l*^3^+ 1.33*l*^2^ - 0.07*l* + 0.39 | 0.94 |
| **Knee**  **Moment** | KMx1 | y = -0.25*l* + 0.06 | 0.73 | y = -0.02*l*^2^ - 0.2*l* + 0.04 | 0.77 | y = 0.28*l*^3^- 0.84*l*^2^ + 0.56*l* - 0.19 | 0.77 |
|  | KMx2 | y = 0.75*l* - 0.47 | 0.72 | y = 0.86*l*^2^ - 0.96*l* + 0.32 | 0.89 | y = -0.41*l*^3^+ 2.06*l*^2^ - 2.08*l* + 0.65 | 0.89 |
|  | KMx3 | y = -0.16*l* - 0.12 | 0.48 | y = -0.18*l*^2^ + 0.19*l* - 0.28 | 0.54 | y = -0.29*l*^3^+ 0.68*l*^2^ - 0.61*l* - 0.05 | 0.54 |
|  | KMx4 | y = 0.14*l* - 0.02 | 0.66 | y = 0.09*l*^2^ - 0.04*l* + 0.06 | 0.73 | y = -0.2*l*^3^+ 0.69*l*^2^ - 0.6*l* + 0.23 | 0.73 |
| **Hip**  **Moment** | HMx1 | y = 0.82*l* - 0.38 | 0.82 | y = 0.66*l*^2^ - 0.49*l* + 0.23 | 0.94 | y = -0.21*l*^3^+ 1.28*l*^2^ - 1.07*l* + 0.41 | 0.94 |
|  | HMx2 | y = -0.73*l* + 0.27 | 0.87 | y = -0.45*l*^2^ + 0.16*l* - 0.15 | 0.95 | y = -0.11*l*^3^- 0.14*l*^2^ - 0.13*l* - 0.06 | 0.96 |
|  | HMx3 | y = 0.42*l* - 0.22 | 0.84 | y = 0.25*l*^2^ - 0.08*l* + 0.01 | 0.93 | y = -0.4*l*^3^+ 1.42*l*^2^ - 1.17*l* + 0.33 | 0.93 |
| **Ankle**  **Power** | APx1 | y = -0.62*l* + 0.39 | 0.77 | y = -0.73*l*^2^ + 0.83*l* - 0.28 | 0.95 | y = -0.71*l*^3^+ 1.35*l*^2^ - 1.11*l* + 0.3 | 0.95 |
|  | APx2 | y = -0.89*l* + 0.24 | 0.81 | y = 1.25*l*^2^ - 3.37*l* + 1.39 | 0.84 | y = 3.01*l*^3^- 7.63*l*^2^ + 4.92*l* - 1.07 | 0.88 |
|  | APx3 | y = 4.49*l* - 2.77 | 0.86 | y = 3.31*l*^2^ - 2.09*l* + 0.27 | 0.98 | y = -3.69*l*^3^+ 14.19*l*^2^ - 12.24*l* + 3.28 | 0.98 |
| **Knee**  **Power** | KPx1 | y = 0.65*l* - 0.44 | 0.72 | y = 0.73*l*^2^ - 0.79*l* + 0.22 | 0.90 | y = -0.6*l*^3^+ 2.5*l*^2^ - 2.45*l* + 0.71 | 0.90 |
|  | KPx2 | y = -1.1*l* + 0.76 | 0.64 | y = -1.79*l*^2^ + 2.46*l* - 0.88 | 0.91 | y = -0.91*l*^3^+ 0.9*l*^2^ - 0.05*l* - 0.14 | 0.93 |
|  | KPx3 | y = 0.67*l* - 0.33 | 0.80 | y = 0.39*l*^2^ - 0.1*l* + 0.03 | 0.87 | y = 0*l*^3^+ 0.39*l*^2^ - 0.1*l* + 0.03 | 0.87 |
|  | KPx4 | y = -1.08*l* + 0.59 | 0.83 | y = -0.78*l*^2^ + 0.46*l* - 0.13 | 0.93 | y = 0.26*l*^3^- 1.54*l*^2^ + 1.17*l* - 0.34 | 0.93 |
| **Hip**  **Power** | HPx1 | y = 0.81*l* - 0.44 | 0.77 | y = 0.47*l*^2^ - 0.13*l* - 0.01 | 0.84 | y = 0.07*l*^3^+ 0.26*l*^2^ + 0.07*l* - 0.06 | 0.84 |
|  | HPx2 | y = -0.65*l* + 0.39 | 0.77 | y = -0.79*l*^2^ + 0.91*l* - 0.33 | 0.95 | y = -0.9*l*^3^+ 1.88*l*^2^ - 1.57*l* + 0.4 | 0.96 |
|  | HPx3 | y = 1.03*l* - 0.56 | 0.87 | y = 0.63*l*^2^ - 0.22*l* + 0.02 | 0.95 | y = -0.49*l*^3^+ 2.06*l*^2^ - 1.55*l* + 0.41 | 0.95 |

Stride length (*l*)
